# Preliminary Transcriptome Analysis of Mature Biofilm and Planktonic Cells of *Salmonella* Enteritidis Exposure to Acid Stress

**DOI:** 10.3389/fmicb.2017.01861

**Published:** 2017-09-26

**Authors:** Kun Jia, Guangyu Wang, Lijiao Liang, Meng Wang, Huhu Wang, Xinglian Xu

**Affiliations:** ^1^National Center of Meat Quality and Safety Control, Nanjing Agricultural University, Nanjing, China; ^2^Jiangsu Collaborative Innovation Center of Meat Production and Processing, Quality and Safety Control, Nanjing Agricultural University, Nanjing, China

**Keywords:** *Salmonella* Enteritidis, biofilm, acid stress, transcriptome, sRNAs

## Abstract

*Salmonella* has emerged as a well-recognized food-borne pathogen, with many strains able to form biofilms and thus cause cross-contamination in food processing environments where acid-based disinfectants are widely encountered. In the present study, RNA sequencing was employed to establish complete transcriptome profiles of *Salmonella* Enteritidis in the forms of planktonic and biofilm-associated cells cultured in Tryptic Soytone Broth (TSB) and acidic TSB (aTSB). The gene expression patterns of *S*. Enteritidis significantly differed between biofilm-associated and planktonic cells cultivated under the same conditions. The assembled transcriptome of *S*. Enteritidis in this study contained 5,442 assembled transcripts, including 3,877 differentially expressed genes (DEGs) identified in biofilm and planktonic cells. These DEGs were enriched in terms such as regulation of biological process, metabolic process, macromolecular complex, binding and transferase activity, which may play crucial roles in the biofilm formation of *S*. Enteritidis cultivated in aTSB. Three significant pathways were observed to be enriched under acidic conditions: bacterial chemotaxis, porphyrin-chlorophyll metabolism and sulfur metabolism. In addition, 15 differentially expressed novel non-coding small RNAs (sRNAs) were identified, and only one was found to be up-regulated in mature biofilms. This preliminary study of the *S*. Enteritidis transcriptome serves as a basis for future investigations examining the complex network systems that regulate *Salmonella* biofilm in acidic environments, which provide information on biofilm formation and acid stress interaction that may facilitate the development of novel disinfection procedures in the food processing industry.

## Introduction

Salmonellosis is a worldwide public health issue due to its increasing hospitalization and mortality rates in many countries (Scallan et al., 2013; Ziebell et al., 2017). The genus *Salmonella* comprises more than 2,500 serovars that have been associated with animal or human infection, and *Salmonella enterica* subspecies *enterica* serovar Enteritidis is the most common serovar in food outbreaks and infection (Carrique-Mas et al., 2008; Vieira et al., 2009; Sangal et al., 2010; Ziebell et al., 2017). According to the 2016 EFSA-ECDC annual report, *S*. Enteritidis was confirmed in 31,829 (45.7%) cases of salmonellosis in humans in 2015 (EFSA-ECDC, 2016), and the majority of salmonellosis is caused by *S*. Enteritidis in most developed countries (Ashton et al., 2016).

*Salmonella* can colonize processing surfaces and equipment such as stainless steel, marble and granite, and traditional cleaning and sanitation procedures may not be able to eradicate *Salmonella* from such surfaces (Rodrigues et al., 2011; Arguello et al., 2012). *Salmonella* is usually present on the processing surfaces of food equipment in the form of a biofilm, which can enable cells to cope with harsh conditions, in particular the Typhimurium and Enteritidis serotypes (Simões et al., 2010; Finn et al., 2013; Wang et al., 2015), and cells present in the biofilm matrix are more resistant to routinely used disinfectants than their planktonic cells (Joseph et al., 2001; Soni et al., 2013). Recent studies have shown that *Salmonella* species are able to survive under various environmental conditions, being found on poultry, meat, melons and grains as well as in the food processing industry (Bower and Daeschel, 1999; Stepanovi et al., 2004).

Many studies have assessed the responses of planktonic bacteria or formed-biofilms to environmental stresses such as acid, sodium chloride, starvation, and heat (Scher et al., 2005; Rodrigues et al., 2011; O'Leary et al., 2015; Philips et al., 2017). Previous studies have shown that acid tolerance, an adaptive system organisms use to respond to mild acidic conditions, can enable *Salmonella* to survive more extreme acidic conditions (Foster and Hall, 1990; Foster, 1995). It has been demonstrated that *Salmonella* Typhimurium cells exposed to mild acidic conditions of pH 4.5–5.8 can survive more extreme acidic conditions through the acid tolerance response (ATR) as time goes by Foster (1995), Bang et al. (2000). Wang et al. (2016) found that long-term acid stress obviously inhibited the biofilm formation of *S*. Enteritidis within a pH range of 5.0–7.2 compared with a control group (pH 7.2); however, no difference was observed in planktonic cell numbers. Several studies have focused on the resistance of biofilm formation to acid stress at different time points, such as short-term or long-term acid stress (Kubota et al., 2008; Chen et al., 2015; Wang et al., 2016). However, the molecule mechanisms underlying the gene expression networks that function during biofilm formation remain unclear, in particular the small non-coding RNAs (sRNAs). Whether the sRNAs of *S*. Enteritidis were involved in the stress tolerance or the regulation of stress response is not clear. Therefore, a deeper understanding of these mechanisms is necessary for the development of effective biofilm removal methods in the food industry.

High-throughput deep sequencing, sequencing hundreds of thousands to millions of DNA molecules at one time, is one of the latest techniques used to investigate the global gene expression profiles of pathogenic bacteria in response to environmental stress (Karavolos et al., 2008; Deng et al., 2012; Amin et al., 2016; Beaubrun et al., 2017; Philips et al., 2017). A number of studies have evaluated the resistance of *Salmonella* planktonic cells to various stresses, in particular of *Typhimurium* serovar (Mühlig et al., 2014; Amin et al., 2016; Ryan et al., 2016), however, few study have focused on the transcriptome of biofilm formed by *Enteritidis* serovar exposed to acid stress due to the incomplete reference genome. Therefore, the goal of this study was to study the global gene expression profiles of mature biofilms formed by *S*. Enteritidis on a stainless steel surface by RNA-seq in acidic TSB (pH = 6.0 TSB) and regular culture medium (pH = 7.2 TSB) compared with planktonic cells.

## Materials and methods

### Bacterial strain and culture conditions

The *S*. Enteritidis (NMC1461) strain used in this study was isolated from meat processing surfaces in 2014 and was stored in National Center of Meat Quality and Safety Control in China. The strain was stored frozen (−80°C) in 40% glycerol before utilization and was twice resuscitated by cultivation in tryptic soy agar (TSA, Luqiao Technology Co. Ltd., Beijing, China). A single colony of the strain was then inoculated into Tryptic Soytone Broth (TSB, Luqiao Technology Co. Ltd., Beijing, China) at 37°C for 18 h, and cells from the stationary phase were harvested by centrifugation at 10,000 × g for 10 min at 4°C, washed twice with 10 mL of an 0.85% (*w/v*) NaCl solution and finally re-suspended in an 0.85% NaCl solution (ca. 10^8^ CFU/ml) and used as the inoculum for the biofilm formation assay.

### Biofilm formation and acid treatment

Food grade stainless steel (GSS) coupons (75 × 25 × 1 mm, 304 type, 2b finish, Shunfen Stainless Steel Material Co. Ltd., Tongnan, China) were the non-biological substrates used for biofilm development. Prior to use, the coupons were washed with acetone (overnight) as previously described (Giaouris et al., 2013). To produce biofilms, 20 cleaned GSS coupons were placed in a glass box to ensure that the upper part of each coupon (ca. 12 mm) was exposed to the liquid-air interface, which provides a good environment for biofilm formation. The glass box containing the GSS coupons was then autoclaved at 121°C for 15 min. Sterilized and cooled TSB (pH = 7.2) or acidic TSB (pH = 6.0) (230 ml) with a 230 μl (10^8^ CFU/ml) aliquot of a cell suspension, prepared as described above, was transferred into the cooling glass box containing 20 GSS coupons. The inoculated box was incubated at 20°C for 3 days without any dynamic conditions to facilitate biofilm development on the coupons, and the planktonic cells (grown without stainless steel in TSB or aTSB fluid) were incubated in the same manner. A 0.1 M phosphate buffer solution was added to the TSB to maintain a pH of 6.0 for the acid stress group. Non-attached cells were removed by rinsing the coupons three times with a 0.85% NaCl solution, whereas the attached biofilm cells were detached from the coupons using a violent water-flapping motion that has been proven to effectively remove cells attached to coupons (Wang et al., 2015). The coupons were transferred into a stomacher blender bag and shaken at a speed of 200 oscillations/min for 2 min using a bag mixer (BagMixer 400VW, Interscience) to detach the cells from the coupons. The planktonic cells were harvested by centrifugation (10,000 × g, 15 min) at 4°C, whereas the biofilm cells were harvested by passage through a filter with a pore diameter of 0.22 μm pore.

### Total RNA isolation, library construction, and sequencing

Total RNA for Illumina sequencing was extracted from the biofilm cells (TB, aTB) and the planktonic cells (TF, aTF) prepared as described above using a Trizol Bacterial RNA Isolation Kit (Thermo Fisher) according to the manufacturer's protocol. During RNA isolation, DNA was removed by treating the samples with RNase-free DNase (Solarbio) for 30 min at 37°C to avoid DNA contamination. The purity, concentration and integrity of the RNA was examined using a NanoPhotometer spectrophotometer (IMPLEN, CA, USA), Qubit 2.0 fluorometer (Life Technologies, CA, USA) and an Agilent Bioanalyzer 2100 system (Agilent Technologies, CA, USA). The total RNA in each sample used for library construction had to pass quantity and quality control. The RNA integrity number (RIN) values of all the samples used for RNA-seq were >6.0 (Schroeder et al., 2006).

A total of 3 μg of each RNA sample was used as the input material for the RNA sample preparations, and three biological replicates were analyzed for a total of 12 (3 × 4) samples. The sequencing libraries, in which index codes were added to attribute sequences to each sample, were constructed and sequenced using an NEBNext Ultra™ Directional RNA Library Prep Kit for Illumina (NEB, USA) according to the manufacturer's recommendations. Briefly, mRNA was enriched from total RNA via poly-T oligo-conjugated magnetic beads by depleting rRNA and then fragmented into short fragments using an RNA fragmentation reagent (Life Technologies). First-strand cDNA was synthesized from these cleaved mRNA fragments using random hexamer primers and M-MuL V Reverse Transcriptase (RNaseH-), followed by the synthesis of second-strand cDNA using DNA Polymerase I and RNase H in the presence of dATP, dGTP, dCTP, and dTTP. Exonuclease and polymerase were used to blunt and adenylate the 3′ ends of DNA fragments, and NEBNext Adaptors with hairpin loop structures were ligated to the fragments in preparation for hybridization. The library fragments were purified using the AMPure XP system (Beckman Coulter, Beverly, USA) to preferentially select cDNA fragments 150–200 bp in length. Next, the purified cDNA fragments were enriched via PCR, the products were purified (AMPure XP system), and library quality was assessed on the Agilent Bioanalyzer 2100 system. Finally, the cDNA libraries were sequenced using the Illumina HiSeq 2500 platform, and 150 bp paired-end reads were generated.

### Reads alignment and analysis of differential gene expression

The raw reads were filtered by removing low-quality sequences to obtain clean reads according to the following criteria: (1) removing reads containing poly-N; (2) removing reads containing adapters; and (3) removing low-quality reads. At the same time, the Q20 and Q30 and the GC content of the clean data were calculated to evaluate the sequencing quality, and all the downstream analyses were performed using high-quality clean data. The clean reads were then mapped to the *S*. Enteritidis strain SEJ complete genome (NCBI reference sequence: NZ_CP008928.1) using Bowtie2-2.2.3 (Langmead and Salzberg, 2012). The mapping parameters were set as follows: (1) clean reads total mapped to the reference genome, >70%; (2) clean reads multiple mapped to the reference genome, <10%; and (3) clean reads uniquely mapped to the reference genome, >90%.

To estimate the abundance of each unigene, the FPKM (expected number of Fragments Per Kilobase of transcript sequence per Millions of base pairs sequenced) method was used, which considers the effect of gene length and sequencing depth for the read count at the same time and is currently the most common method used to calculate the log2 (FPKM biofilm/FPKM planktonic) fold change. Differential expression analysis of mature biofilm/planktonic cells (three biological replicates per condition) was performed using the DEGSeq R package (1.18.0), which provided statistical routines to identify differentially expressed genes (DEGs) between the mature biofilm and planktonic cells using a model based on the negative binomial distribution with an absolute value of log2 (fold change) >1 and an adjusted *P*-value threshold of <0.5. According to the gene expression profiles, the Pearson correlation coefficients among the 12 samples were calculated.

### Go and KEGG enrichment analysis of differentially expressed genes

The DEGs were used for Gene Ontology (GO) and KEGG (Kyoto Encyclopedia of Genes and Genomes) enrichment analyses to help further understand the biological functions of the identified genes and significantly enriched metabolic pathways. In this study, both GO terms and KEGG pathways with adjusted *P*-value < 0.05 were considered significantly enriched in DEGs.

### Quantitative RT-PCR analysis

Several genes that showed significant up- or down-regulation were selected for quantitative reverse transcription-PCR (qRT-PCR) validation to determine if gene expression was consistent between qRT-PCR and RNA-seq. Total RNA was isolated as described above, followed by cDNA synthesis using reverse transcriptase. Quantitative PCR (qPCR) was carried out for each sample using SYBR green (Prime Script RT Master Mix; TAKARA, China) methodology and the 7,500 Fast Real-time PCR System (Applied Biosystems, Foster, USA) with a total of 500 ng of RNA as the starting material. The 16S DNA of *Salmonella* was used as an internal gene to normalize the expression of the tested genes. The primers used in this study are listed in supporting information Table S1. Melting curve analysis was used to validate the specificity of primers, and the ΔΔCT method was used to calculate the relative gene expression. The expression level of each target gene was compared with the 16S DNA internal control gene using the 2^−ΔΔCt^ method, and each gene was analyzed at least four times.

## Results

### RNA sequencing and preliminary analysis of the raw data

To obtain a comprehensive overview of the transcriptome profile of *Salmonella* biofilms in response to acid stress, RNA was extracted from four different groups, TB, TF, aTB, and aTF, with three independent biological replicates per group. The RNA used for library construction had to pass quantity and quality control criteria. Twelve libraries were constructed from the four different groups, and raw reads were produced from the 12 libraries using the Illumina sequencing platform. A total of 13–28 million reads per sample were generated by RNA-seq (Table 1). Clean reads were harvested with strict quality control criteria and data filtration, and the average numbers of total clean reads in the four libraries were 22,568,103 (TB), 15,179,200 (aTB), 17,549,117 (TF), and 15532433 (aTF). These reads were mapped to the *S*. Enteritidis strain SEJ complete reference genome. Altogether, 91.41% (TB), 92.86 (aTB), 95.11% (TF), and 90.70% (aTF) of the clean reads were uniquely mapped to the reference genome. Under normal circumstances, if the reference genome is appropriate and there is no contamination in the relevant experiments, the percentage of mapped sequences generated by the experiment should be higher than 70%, with multiple mapped reads accounting for <10%. In this study, over 90% of the clean reads for each sample were mapped to the reference genome, with multiple mapped reads accounting for <5%. Additionally, biological repetition is essential to any biological experiment, and in high-throughput sequencing, the square of the Pearson correlation coefficient should be larger than 0.8; if this is not the case, the reason should be determined or the experiment should be repeated. As shown in supporting information Figure S1, the correlation coefficient diagram illustrates the relationship among the four treatments, with an average coefficient (*R*^2^) of 0.989, 0.981, 0.985, and 0.986 for TB, TF, aTF, and aTB, respectively. These results indicated that the reference genome in our study was very suitable and that the basic RNA-seq data could be used for subsequent bioinformatics analysis.

**Table 1:** RNA-seq data statistics.

**Samples^a^**	**Raw reads**	**Clean reads^b^**	**Clean bases^c^**	**Q20 (%)^d^**	**Q30 (%)^e^**	**Multiple mapped (%)^f^**	**Uniquely mapped (%)^g^**
TB1	22972274	21089902	3.16G	96.93	92.14	4.13	91.47
TB2	22982320	21042592	3.16G	96.85	91.97	4.23	91.29
TB3	27939654	25571816	3.84G	96.86	92.04	4.32	91.48
TF1	17048238	15773838	2.37G	98.19	94.95	1.91	95.45
TF2	21089418	19873194	2.98G	98.13	94.83	2.19	94.67
TF3	18183276	17000320	2.55G	98.24	95.09	2.06	95.21
aTF1	19823808	19034174	2.86G	98.15	94.88	2.44	90.72
aTF2	14603550	14014400	2.1G	98.15	94.86	2.15	90.62
aTF3	14113492	13548724	2.03G	98.21	95.02	2.22	90.75
aTB1	13672844	13538222	2.03G	97.67	93.59	3.28	92.82
aTB2	15780488	15643318	2.35G	98.34	95.3	3.23	92.88
aTB3	16476102	16356060	2.45G	98.32	95.24	3.1	92.89

a *1, 2, and 3 represent three independent biological replicates*.

b *Reads from sequencing after filtering low-quality reads*.

c *The number of clean reads is multiplied by the length and converted to G*.

d *Q20*.

e *Q30 The percentage of bases with a Phred value >20 or 30*.

f *The percentages of multiple mapped reads accounting for the total mapped clean reads*.

g *The percentages of unique mapped reads accounting for the total mapped clean reads*.

### Differentially expressed genes (DEGS) under acid stress

**Figure 1:**
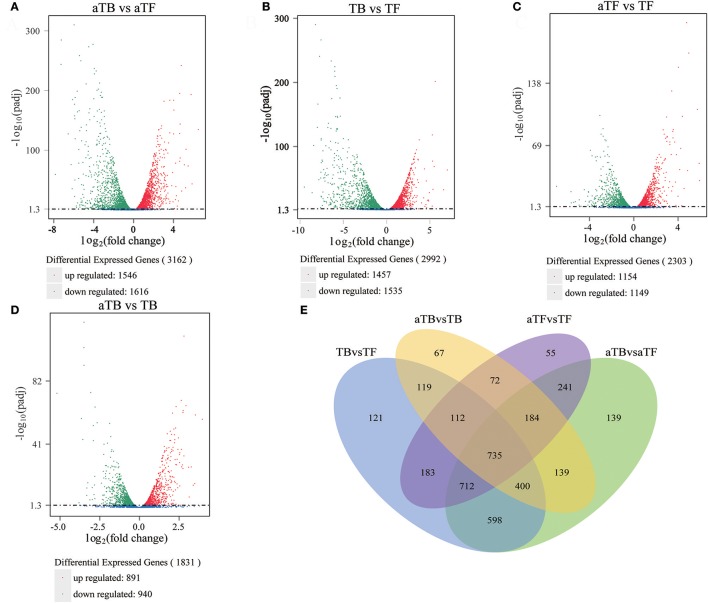
Global comparison of DEGs in different groups: **(A)** Volcano plot of DEGs in aTBF. **(B)** Volcano plot of DEGs in TBF. **(C)** Volcano plot of DEGs in aTFF. **(D)** Volcano plot of DEGs in aTBB. **(E)** Venn diagram of DEGs identified in different groups.

The RNA-seq data were used to evaluate differences in gene expression, and the FPKM was calculated to quantify the expression levels of all genes in the four groups, TB, TF, aTB, and aTF. The DEGs were determined using the DESeq R package with the criteria of log2 (fold change) >1 and adjusted *P*-value <0.05. There were 3,162 genes that were significantly differentially expressed between aTB and aTF, including 1,546 up-regulated genes and 1,616 down-regulated genes (Figure 1A). A total of 2,992 genes were identified between TB and TF, with 1,457 genes up-regulated and 1,535 genes down-regulated (Figure 1B). After acid treatment, 2,303 DEGs were identified in aTFF (aTF vs. TF), and 1,831 DEGs were identified in aTBB (aTB vs. TB). The numbers of DEGs showing up or down-regulation are shown for aTFF (Figure 1C) and aTBB (Figure 1D). The DEGs were classified using a Venn diagram (Figure 1E) which indicated that of the 3,162 DEGs in aTBF and 2,992 DEGs in TBF, 535 genes were specifically altered in TBF, 703 genes were specifically altered in aTBF, and 2,445 genes were differentially expressed in both TBF and aTBF. The DEGs identified in both TBF and aTBF likely play an important role in biofilm formation. Furthermore, there were also 1,103 DEGs identified in aTFF and aTBB, which provided evidence that these DEGs may be induced in an acidic environment and may be essential for the response to acid stress in *Salmonella*.

**Figure 2:**
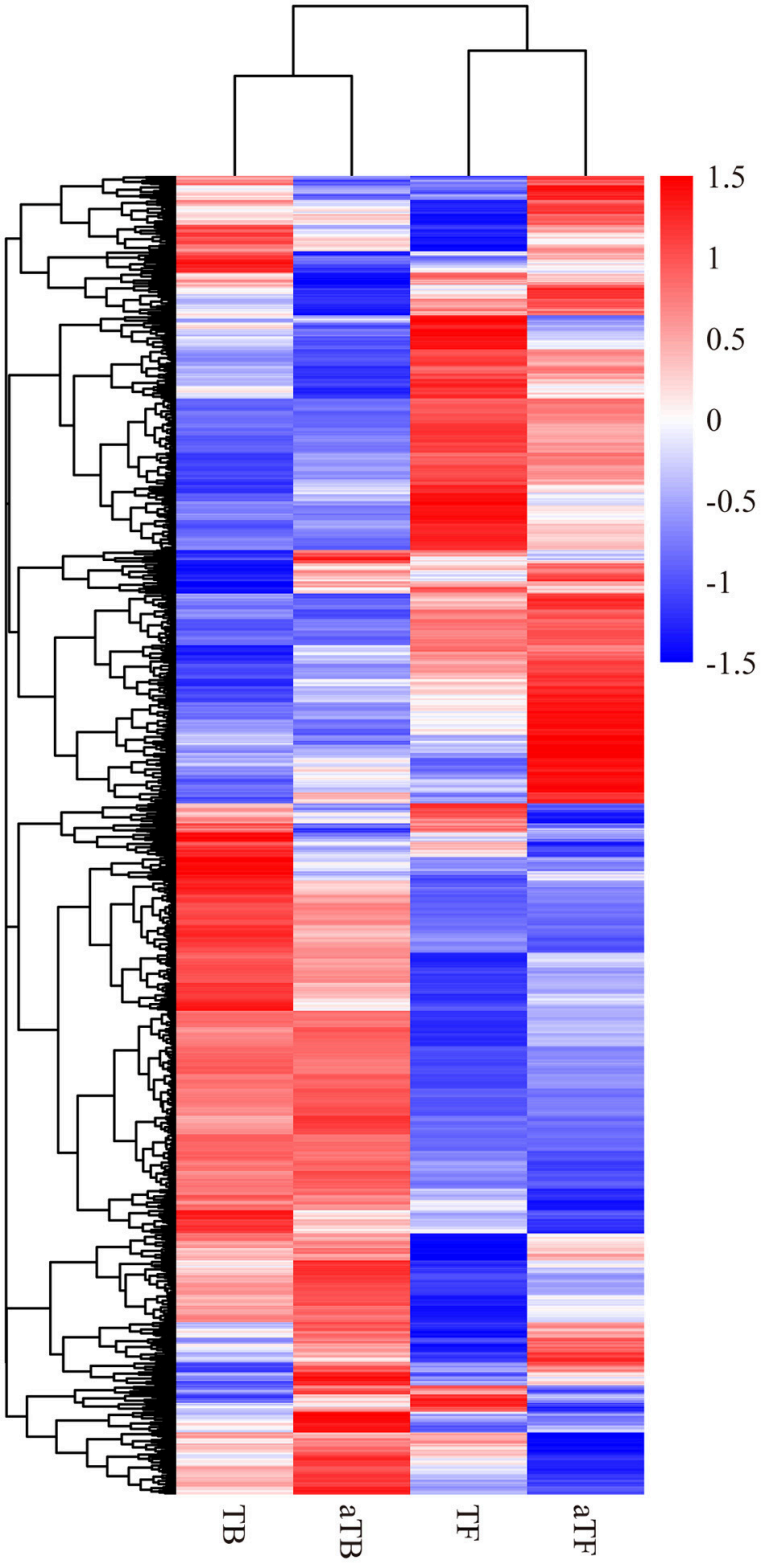
Heatmap and hierarchical clustering of the differentially expressed mRNAs in the four groups: Note: Red indicates a higher mRNA expression level, and blue indicates a lower mRNA expression level. The color from red to blue indicates log10 (FPKM+1) from large to small.

A heat map and hierarchical clustering were used to determine the expression patterns of different genes under different experimental conditions, and genes with similar expression patterns may have similar functions or may be involved in the same metabolic process or cellular pathway. Therefore, the genes with the same or similar expression patterns were clustered into classes to predict the function of unknown genes or new functions of known genes. In this study, FPKM was used to conduct the hierarchical clustering analysis of transcript abundance, and 3,877 DEGs across the four different groups were analyzed. The heat map and hierarchical clustering demonstrated that the TF groups showed similar transcriptome profiles to the aTF groups and that the TB groups were similar to the aTB groups (Figure 2). The biofilm-associated groups (TB, aTB) and planktonic cells groups (TF, aTF) were distinctly separated, and the differentially expressed mRNAs in each group were clustered into two types, one with higher expression in the biofilm-associated groups but lower expression in the planktonic cells groups, and the other with lower expression in the biofilm-associated groups but higher expression in the planktonic cells groups.

### Functional annotation of the transcriptome

**Figure 3:**
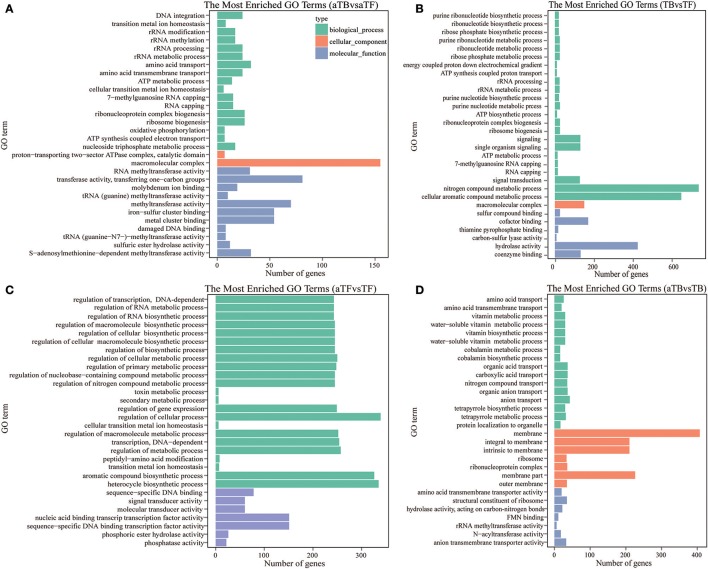
Blast2GO annotation of differentially expressed genes assigned to three GO categories (*p*-value below 0.05). **(A)** The Top 30 Most Enriched Go Terms in aTBF. **(B)** The Top 30 Most Enriched Go Terms in TBF. **(C)** The Top 30 Most Enriched Go Terms in aTFF. **(D)** The Top 30 Most Enriched Go Terms in aTBB.

Gene Ontology (GO) enrichment analysis of DEGs was performed to functionally analyze the *S*. Enteritidis transcriptome according to NR annotation. The GO terms from hierarchical vocabularies describing biological processes, cellular components and molecular functions for analyzing transcripts were assigned using Blast2GO software (Conesa and Götz, 2008). Among the 3,697 genes in aTBF, 2,290 DEGs were assigned to the biological process, cellular component and molecular function categories. These DEGs were assigned to many GO terms, and we selected the 30 most enriched GO terms with *p*-values < 0.05, which are displayed in Figure 3A. The terms “amino acid transport,” “amino acid transmembrane transport,” “macromolecular complex,” “transferase activity, transferring one-carbon groups,” and “iron-sulfur cluster binding” were the most highly represented. Among the 3,697 genes in TBF, 2139 DEGs were assigned to the three GO categories (Figure 3B). The 30 most enriched functional categories included “nitrogen compound metabolic process,” “cellular aromatic compound metabolic process,” “signaling,” “macromolecular complex” and “sulfur compound binding.” There were three biological process terms (metabolic process, biosynthetic process and signaling), one cellular component term (macromolecular complex) and three molecular function terms (binding, methyltransferase activity and transferase activity) that exhibited alterations in both aTBF and TBF, and these categories are likely associated with biofilm formation. As shown in Figure 3C, regulation of biological process played a very important role in the response of *S*. Enteritidis to acid stress, while none of the cellular component terms were significant enriched. The molecular functions of binding, receptor activity and signal transducer activity were also associated with acid stress compared with TF. The top 30 functional categories included acid transport, carboxylic acid transport, membrane part, membrane and transporter activity (Figure 3D).

The KOBAS software was used to test the statistical enrichment of DEGs in KEGG pathways to assess the reliability of our transcriptome results and our annotation process. The 2,139 DEGs in TBF and 2,290 DEGs in aTBF were mapped to 90 different KEGG pathways. However, no significantly enriched pathways were identified in the two groups by KEGG enrichment analysis (*p* < 0.05). As shown in Table 2, three significantly enriched pathways (*p* < 0.05) were identified in aTFF (1 pathway) and aTBB (2 pathways). In the aTBB group, there were a total of 28 DEGs involved in porphyrin and chlorophyll metabolism and 27 DEGs involved in sulfur metabolism. Additionally, a total of 21 DEGs in aTFF were significantly enriched in bacterial chemotaxis. These three significantly enriched pathways may be associated with the acid tolerance of *S*. Enteritidis.

**Table 2:** The numbers of DEGs significantly enriched in KEGG pathways.

	**Pathway**	***P*-value**	**Enrichment factor**	**Number of up-regulated DEGs**	**Number of down-regulated DEGs**
aTF vs. TF	Bacterial chemotaxis	0.047	0.875	6	15
aTB vs. TB	Porphyrin and chlorophyll metabolism	0.038	0.717	22	6
	Sulfur metabolism	0.044	0.711	13	14

Fifteen differentially expressed small non-coding RNAs (sRNAs) were identified in the transcriptomes of the two biofilm-associated groups (Table 3); 11 sRNAs were identified in the TBF group, 10 sRNAs were identified in the aTBF group, and six sRNAs were identified in both groups. Most of the 15 sRNAs were located on the negative DNA strand (Minus), and only three sRNAs (*sRNA314, sRNA282*, and *sRNA290*) were located on the positive DNA strand (Plus). Additionally, 10 of the 15 sRNAs possessed a simple sRNA structure predicted viaMfold (HP), and only five possessed more than one hairpin (HS).

**Table 3:** Differently expressed novel non-coding small RNAs (sRNAs) in the transcriptomes of the two biofilm-associated groups.

**Groups**	**Regulation**	**Name**	**Strand^a^**	**Structure^b^**	**Length**	**Log2 FoldChange**	***Q*-value**
TB vs. TF	Down	*00068*	Minus	HP	57	−5.8575	4.21E^−05^
		*00011*	Minus	HP	63	−3.8289	0.00088
		*00314*	Plus	HP	130	−3.7709	4.65E^−12^
		*00171*	Minus	HS	107	−3.2061	0.00521
		*00282*	Plus	HP	100	−2.9219	1.69E^−10^
		*00236*	Minus	HP	61	−2.2472	1.92E^−06^
		*00183*	Minus	HS	133	−1.6289	3.39E^−10^
		*00123*	Minus	HP	60	−1.5535	4.38E^−12^
		*00109*	Minus	HS	217	−1.4922	0.00019
		*00110*	Minus	HP	61	−1.0064	1.91E^−09^
	Up	*00290*	Plus	HP	78	1.4864	0.00010
aTB vs. aTF	Down	*00218*	Minus	HP	66	−5.1128	0.00696
		*00171*	Minus	HS	107	−3.208	0.00230
		*00290*	Plus	HP	78	−3.1779	3.02E^−14^
		*00282*	Plus	HP	100	−2.8388	4.34E^−22^
		*00236*	Minus	HP	61	−2.4646	1.08E^−07^
		*00237*	Minus	HS	96	−2.4572	1.20E^−12^
		*00314*	Plus	HP	130	−1.7717	0.00830
		*00221*	Minus	HP	104	−1.593	0.00018
		*00103*	Minus	HS	97	−1.509	0.02214
		*00123*	Minus	HP	60	−1.0206	3.25E^−06^

a *Strand:Minus, negative DNA strand; Plus, positive DNA strand*.

b *Structure:Hairpin (HP), simple sRNA structure predicted via Mfold; Highly structured (HS), possesses more than one hairpin*.

### Validation of DEGs

**Figure 4:**
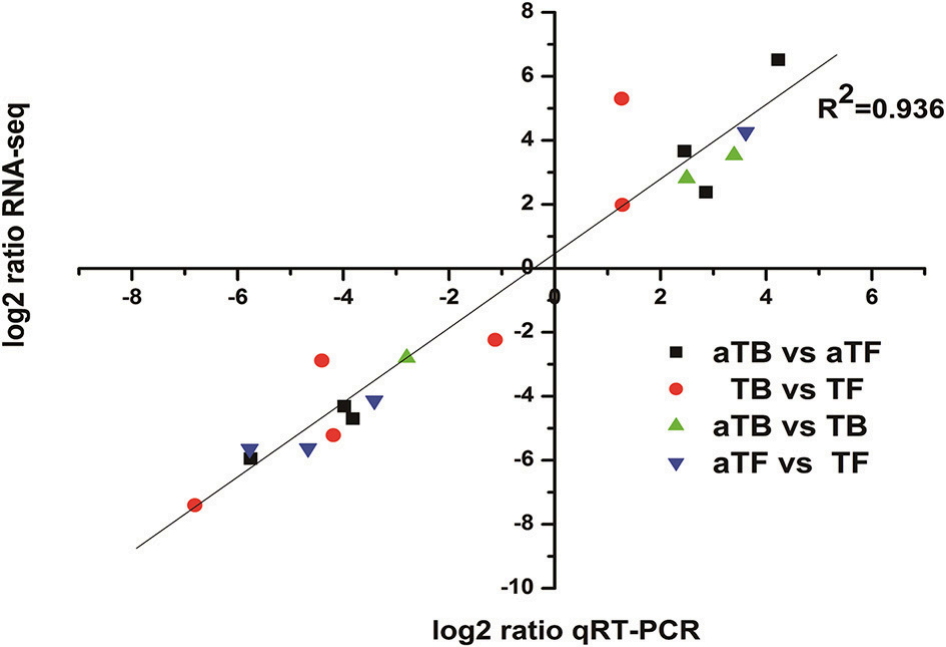
Correlation of gene expression measurements by RNA-seq and qRT-PCR for 19 selected genes. Fold change values were converted to log2 values for comparison.

qRT-PCR was performed on 19 selected genes (8 up-regulated and 11 down-regulated) to verify the RNA-seq data in this study. As shown in Figure 4, the qRT-PCR data correlated well with the RNA-seq data in identifying gene up- or down-regulation (*R*^2^ = 0.936).

## Discussion

Biofilm formation and the bacterial response to acid stress are governed by many regulators, not just several specific genes (Quinn et al., 2014; Wang et al., 2015; Anbalagan et al., 2016). However, the transcriptome and regulatory network involved in biofilm formation or the bacterial stress response in *S*. Enteritidis are only partly known, as most recent studies have focused on the transcriptome of *S*. Typhimurium (Wang et al., 2015; Anbalagan et al., 2016), while few studies have focused on *S*. Enteritidis (Shah, 2014). Therefore, it is critical to identify the transcriptome profile of *S*. Enteritidis. In the present study, RNA-seq technology was used to obtain the transcriptome profile of mature *S*. Enteritidis biofilms on a stainless steel surface subjected to acid stress. Furthermore, we used comparative transcriptomics to compare biofilm cells with planktonic cells and found that thousands of genes were specifically up- or down-regulated. Additionally, we identified several novel sRNAs and performed functional analyses (KEGG, GO annotation). The expression levels determined by qRT-PCR were consistent with the results from RNA-seq. The following aspects associated with acid stress or biofilm formation were characterized from a global perspective.

### DEGs identified by comparing biofilm-associated cells with planktonic cells

Our results showed that thousands of genes were significantly up- or down-regulated in biofilm-associated cells compared with planktonic cells. Of these genes, 20 that were differentially expressed and likely had an impact on biofilm formation are shown in Table 4. The *fimFH* genes showed high levels of expression, and FimH protein in particular has been the focus of many studies. In a previous study, FimH protein was found to be the mannose-specific adhesin of *Escherichia coli* type 1 fimbriae (Krogfelt et al., 1990), and *E. coli* biofilm formation could be significantly reduced by inhibition of FimH (Sarkar et al., 2016). It has been shown that the minor fibril components FimF, FimG, and FimH are involved in receptor binding, which is important for the initial attachment. In the present study, the expression change (more than 3-fold) of *fimFH* in TBF was similar to that observed in aTBF, showing significant up-regulation in biofilm-associated cells. A similar finding has also been observed by Pratt and Kolter (1998), who presented evidence that FimH protein was required for initial surface attachment. Compared with planktonic cells, the *prpDE* gene involved in propanoate metabolism was highly expressed in biofilm cells in our study. Consistent with our result, *prpBCDE* expression was found to be higher in biofilm-associated cells of *E. coli* O157:H7 compared with planktonic cells (Lee et al., 2011). Additionally, Lee et al. (2007) suggested that *prpCDE*, which induces *E. coli* biofilm formation, is involved in propanoate metabolism. The regulation of the prp operon is complex due to the transcriptional regulators, such as PrpR, the σ^54^ factor RpoN, and IHF, that are required to activate the transcription of *prpBCDE* (Simonte et al., 2017). The RNA polymerase sigma factor RpoS, which is required for virulence, stress resistance and biofilm formation, was found to be up-regulated in biofilm-associated cells in this study. RpoS can bind to the *csgD* promoter and has an important impact on the transcription of *csgD*, which is required for the formation of biofilms and production of cellulose by regulating the aggregation and motility of cells (Gerstel et al., 2003; Jonas et al., 2007). In addition, hundreds of hypothetical proteins were differentially expressed in both TBF and aTBF, indicating that they are likely important in biofilm formation. The differentially expressed hypothetical proteins are shown in Table 4.

**Table 4:** DEGs (up- or down-regulated) in different groups.

	**Name**	**Length**	**Log2 Fold Change**	***Q*-value**	**Product or description**
TB vs. TF	*fimH*	1008	5.3012	1.39E^−117^	Adhesin
	*fimF*	519	4.3905	1.68E^−64^	Adhesin
	*prpD*	1452	3.0211	1.24E^−40^	2-methylcitrate dehydratase
	*prpE*	1887	2.9446	6.61E^−43^	Propionate–CoA ligase
	*RS06450*	993	1.9846	1.45E^−34^	RNA polymerase sigma factor RpoS
	*RS02175*	891	−8.4037	4.66E^−101^	Hypothetical protein
	*RS21920*	555	−8.1927	1.44E^−289^	Hypothetical protein
	*RS02245*	1272	−8.1116	0	Hypothetical protein
	*fliA*	720	−4.3271	4.90E^−37^	RNA polymerase sigma factor FliA
	*flgK*	1662	−3.2153	3.52E^−25^	Flagellar hook-associated protein 1
aTB vs. aTF	*prpD*	1452	5.8943	1.78E^−44^	2-methylcitrate dehydratase
	*prpE*	1887	5.2302	7.25E^−38^	Propionate–CoA ligase
	*fimH*	1008	3.6608	4.75E^−130^	Adhesin
	*fimF*	519	3.3435	1.076E^−43^	Adhesin
	*RS06450*	993	2.3788	1.76E^−77^	RNA polymerase sigma factor RpoS
	*RS02245*	1272	−7.2526	9.40E^−245^	Hypothetical protein
	*RS02175*	891	−6.2471	7.40E^−77^	Hypothetical protein
	*RS02240*	549	−6.1948	0	Hypothetical protein
	*fliA*	720	−2.3034	8.24E^−05^	RNA polymerase sigma factor FliA
	*flgk*	1662	−1.4816	8.66E^−40^	Flagellar hook-associated protein 1
aTF vs. TF	*RS03030*	282	4.254	8.84E^−67^	Outer membrane protein
	*RS06065*	411	3.7112	9.30E^−74^	Formate hydrogenlyase maturation protein HycH
	*hycI*	471	3.2794	9.20E^−74^	Hydrogenase 3 maturation endopeptidase HycI
	*rbsC*	966	2.8462	1.51E^−32^	Ribose ABC transporter permease
	*RS06100*	462	2.644	4.19E^−51^	Formate hydrogenlyase regulatory protein HycA
aTB vs. TB	*RS06320*	1119	2.813	3.45E^−34^	Invasion protein InvE
	*RS00680*	327	2.7514	5.43E^−26^	Type III secretion system chaperone SseA
	*RS00655*	417	2.4205	1.68E^−13^	Pathogenicity island 2 effector protein SseE
	*RS00665*	1455	2.3799	5.26E^−16^	Pathogenicity island 2 effector protein SseC

### Up-regulated genes in response to acid stress

As shown in Table 4, genes associated with formate hydrogenlyase, (such as *RS06065, HycI, RS06100, HycE*, and *HycE*), ABC transporter permease (*rbsC*) and outer membrane proteins (*RS03030*) were up-regulated in aTF compared with TF. The *HycADEHI* genes were up-regulated, with a log2 fold change of more than 2 in aTF compared with TF. The *Hyc* genes, which are involved in the cellular metabolism of *S*. Typhimurium, can remove excess reductant generated during acid stress by producing H_2_ and consuming protons, so the expression of hydrogenase is likely involved in the pH response and regulation (Zbell and Maier, 2009; Noguchi et al., 2010). HycA is thought to regulate the Hyc operon, HycD is a transmembrane protein, HycE is the Ni-containing large subunit of Hyc-hydrogenase, and HycI is known to play an important role in the maturation of the formate hydrogenlyase (FHL) complex (Lamichhane-Khadka et al., 2015). In the present study, the *HycADEHI* genes were up-regulated in aTF, with log2 fold changes of 2.1 to 3.7, and all of these genes are associated with hydrogenase. Consistent with our results, Noguchi et al. (2010) observed that hydrogenase-3, known as a part of the FHL complex, contributed to acid resistance of anaerobic cultures by consuming protons. A similar result was found by Hayes et al. (2006), who suggested that hydrogenase plays an important role in acid resistance in partly aerated cultures. A number of genes associated with ABC transporters showed significant up- or down-regulation in aTF compared with TF, reflecting the changing requirements for cells when encountering an acidic environment. The D-ribose transporter gene *rbsC* is one such gene, showing significant up-regulation under aTF in this study. A previous study showed that molecular pumps, characterized as ATP-binding cassette (ABC) transporters, translocate solutes across the membrane by utilizing the energy of ATP hydrolysis (Oldham et al., 2013), and several other bacterial ABC transporters changed significantly after acid shock (Bore et al., 2007). Additionally, levels of the *rbsABCD* operon, which encodes D-ribose transporter components, were up-regulated (7.0–14.96-fold) during artificial gastric fluid stress (Sun et al., 2014).

Of particular interest were genes associated with virulence in the present study, whose levels were mostly up-regulated under aTB compared with TB. As shown in Table 4, the protein InvE, which is also encoded within SPI-1, is necessary for translocating bacterial proteins into host cells and dramatically up-regulated in aTB in this study, with a log2 fold change of 2.8. Quinn et al. (2014) found that when *S*. Typhimurium was subjected to acid stress, which is induced for optimal expression of its virulence genes. In contrast to our results, the *invE* gene was down-regulated in lysogenic *S*. Typhimurium treated at pH 3, 4, and 5 for a short time (Kim et al., 2014). In addition, several proteins associated with virulence, such as SseA, SseE and SseC, were over-expressed in aTB compared with TB. Several studies have shown that virulence factors (called effectors), which are the matrix of the secretory device, encoded by SPI-2 play an important role in the pathogenicity of *Salmonella* (Li et al., 2015; Sun et al., 2016). The secretion activity of SPI-2 is probably induced in response to an acidic environment, and Coombes et al. (2004) demonstrated that the expression of SPI-2-encoded effectors is acid-regulated. In our study, the expression of most effector proteins was up-regulated under aTB. Consistent with our results, higher expression of genes associated with virulence and invasion was found during biofilm formation at pH 5 compared with pH 7 (O'Leary et al., 2015).

### Prediction of sRNAs differentially expressed in biofilm-associated cells

According to our RNA-seq data, a total of 88 candidate sRNAs identified in our study need to be further investigated. Of note, only 15 of these sRNAs were differentially expressed (|log2 fold change| > 1) under TBF and aTBF, and only one of the sRNAs showed up-regulation in biofilm-associated cells compared with planktonic cells. Small RNAs (sRNAs), short non-coding RNA sequence with transcript sizes ranging from 50 to 500 nucleotides, are ubiquitous regulators that play an important role in many cellular activities, affecting the translation of mRNAs by imperfect base pairing with the help of Hfq (Huang et al., 2009; Storz et al., 2011). Large numbers of sRNAs acting as major posttranscriptional regulators are induced in different physiological environments. Jørgensen et al. (2013) observed that CsgD is linked to biofilm formation by regulating the transcription of curli biogenesis under the control of multiple sRNAs (*GcvB, RprA, OmrA, OmrB*). In our study, to determine whether our 88 predicted sRNAs resemble known bacterial sRNAs, we aligned the sequences of our sRNAs to sequences in 3 non-coding RNA databases: Rfam, sRNATarBase and BSRD (Amin et al., 2016). To our surprise, only 10 of our 88 candidate sRNAs aligned significantly with known sRNAs (supporting information: Table S2), including *RyfA, GcvB, Sib*, and *GlmZ*, via comparative analyses with the chromosomes of *Salmonella, E. coli* and *Shigella flexneri* with the help of Hfq RNA binding protein, and none of the 15 differentially expressed sRNAs exhibited significant identity to known sRNAs, likely because this is the first report to investigate the sRNAs associated with biofilm formation in *S*. Enteritidis. Importantly, this means that 78 of the 88 sRNAs in our study are novel and characteristic in *S*. Enteritidis. Additionally, six sRNAs were down-regulated in both TBF and aTBF, which indicated that these six sRNAs probably play prominent role in biofilm formation. However, this hypothesis needs to be further validated using gene knockout technology, such as λ-red homologous recombination and CRISPR-Cas9 (Song et al., 2015).

## Conclusion

*Salmonella* biofilm is considered to be a common source of cross-contamination, posing a serious threat to food factories worldwide where various environmental stresses are encountered. The RNA-seq data in this study provide insight into the gene expression patterns, gene functional categories and significant pathways associated with acid stress or biofilm formation. Furthermore, the list of 15 novel differentially expressed sRNAs predicted in this study provides candidates for further studies. This global analysis of the *S*. Enteritidis transcriptome revealed new candidate genes that will increase our knowledge of the *S*. Enteritidis response to acid stress and may suggest ways to prevent biofilm cross-contamination, bringing us one step closer to uncovering the complex gene networks and molecular mechanisms that involved in the regulation of mature biofilms or acid stress.

## Author contributions

HW and XX designed research; KJ, GW, LL, HW, and MW performed research; KJ and HW wrote the paper.

### Conflict of interest statement

The authors declare that the research was conducted in the absence of any commercial or financial relationships that could be construed as a potential conflict of interest.
