# Supplementary material for: Preliminary Transcriptome Analysis of Mature Biofilm and Planktonic Cells of Salmonella Enteritidis Exposure to Acid Stress
Source: Front Microbiol. 2017 Sep 26;8:1861. doi: 10.3389/fmicb.2017.01861 (PMC5622974; doi:10.3389/fmicb.2017.01861)
Supplement: Supplementary file 1 [file Table1.pdf]

Table S1 Primer pairs used for qRT-PCR in this study

GeneID	Gene/protein annotation	Primer pair
16s DNA	Transcription	F, 5’-AGGCCTTCGGGTTGTAAAGT-3’<br>R, 5’-GTTAGCCGGTGCTTCTTCTG-3’
RS22505	transcriptional regulator	F,5’-GGCGGCATTCCGGCGATAAT-3’<br>R,5’-TGTTGCATCCATGGCCGGTG-3’
RS00885	AraC family transcriptional regulator	F,5’-ACGGAAACCACCGGACAGGA-3’<br>R,5’-AGCAATTCGCCGGCTACCAC-3’
RS12810	PTS sugar transporter	F,5’-AGCATCATGCCGCCCATTCC-3’<br>R,5’-TACGCCGGGCCTGCTCATTA-3’
RS06450	RNA polymerase sigma factor RpoS	F,5’-GCGAATCCACCAGGTTGCGT-3’<br>R,5’-GTGGACTGGCGTTGCTGGAC-3’
RS17410	adhesin	F,5’-GGTGACGATCCCTCGCCAGA-3’<br>R,5’-TCCACGACCTGTCCGGCATT-3’
RS05790	NAD-dependent succinate-semialdehyde dehydrogenase	F,5’-TCGTTTACCGGCTCGACGGA-3’<br>R,5’-CGCGCCTTCGACGGCTTTAT-3’
RS16605	diguanylate cyclase AdrA	F,5’-CTGTCTTTGGCGGCTGGTGG-3’<br>R,5’-ACATGCCGGAGAGGATCGCA-3’
RS10680	protein MgtC	F,5’-CTCGGCGGATACCGGGAGTT-3’<br>R,5’-CATCGGCGTGTTATGCGGCT-3’
RS13320	AraC family transcriptional regulator	F,5’-TGGGGCCGAAAAGTCTGCAT-3’<br>R,5’-ACGACATCGTCCGGGGAGTA-3’
RS14135	fimbrial protein	F,5’-AGCCACTGTCCCGTTCGTTG-3’<br>R,5’-ACCTGCAAGCCCGTCAATTCC-3’
RS06320	invasion protein InvE	F,5’-CTCCAGGCGCGAACAACTGG-3’<br>R,5’-GGCTTATTGCGCGCCAGCTA-3’
RS17740	citrate (pro-3S)-lyase subunit beta	F,5’-CAGCAGTTCGGTGCCTTCCG-3’<br>R,5’-CGGTAGAAATCGCCCACGCC-3’
RS12125	sulfate transporter subunit	F,5’-ACGATCAGGCCAAAGCGCAG-3’<br>R,5’-GTCGCCAGTAGCGCTTCGTT-3’
RS03030	outer membrane protein	F,5’-CACCTCTCATCGGCGTAGCG-3’<br>R,5’-GCGCCAACGTGCAGTACAGA-3’
RS00635	type III secretion system needle protein SsaG	F,5’-AGGCAAATTGCGCTTTAATCATCG-3<br>R,5’-GGATATGCTCTCCCACATGGCG-3’
RS02025	minor curlin subunit	F,5’-TCCTTCCTGGCGTACTCTGGC-3’<br>R,5’-CTGGGATTGCAACCGCGACA-3’
RS11700	3-ketoacyl-CoA thiolase	F,5’-GAATGCGCCAGACTGGGTGG-3’<br>R,5’-ATGATGGGGCTAACGGCGGA-3’
