# Supplementary material for: Preliminary Transcriptome Analysis of Mature Biofilm and Planktonic Cells of Salmonella Enteritidis Exposure to Acid Stress
Source: Front Microbiol. 2017 Sep 26;8:1861. doi: 10.3389/fmicb.2017.01861 (PMC5622974; doi:10.3389/fmicb.2017.01861)
Supplement: Supplementary file 2 [file Table2.pdf]

Table S2 Significant alignment with known sRNAs

sRNA name	Strand	Length	Known sRNA name in bacteria	Structure
sRNA00048	Plus	247	*S.* Typhimurium, *Escherichia coli, Shigella flexneri*, RyfA	HS
sRNA00073	Minus	210	*S.* Typhimurium, *Escherichia coli,* GcvB	HS
sRNA00078	Plus	268	*S.* Typhimurium, *Escherichia coli,* Sib	HS
sRNA00128	Plus	159	*S.* Typhimurium, *Escherichia coli,* GlmZ	HP
sRNA00132	Plus	51	*S.* Typhimurium, *Escherichia coli, S.* Typhimurium, *Yersinia pseudotuberculosis,* SPOT42	HP
sRNA00180	Minus	52	*S.* Typhimurium, sroC	HP
sRNA00187	Plus	67	*S.* Typhimurium, *Enterobacteria*, RtT	HP
sRNA00273	Plus	247	*S.* Typhimurium, *Shigella flexneri*, RyfA	HS
sRNA00287	Plus	268	*S.* Typhimurium, *Escherichia coli,* Sib	HS
sRNA00301	Plus	159	*S.* Typhimurium, *Escherichia coli,* GlmZ	HP
