# Supplementary material for: Preliminary Transcriptome Analysis of Mature Biofilm and Planktonic Cells of Salmonella Enteritidis Exposure to Acid Stress
Source: Front Microbiol. 2017 Sep 26;8:1861. doi: 10.3389/fmicb.2017.01861 (PMC5622974; doi:10.3389/fmicb.2017.01861)
Supplement: Supplementary file 3 [file Image1.pdf]

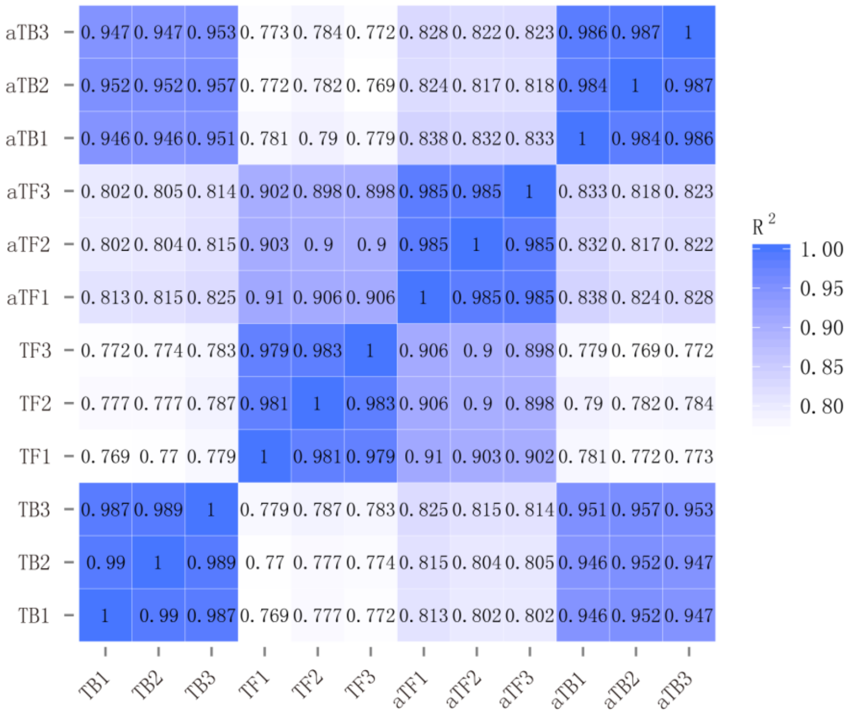

FIG S1 The correlation coefficients among the twelve RNA-seq libraries (correlation matrix of the whole data set).
